# The unmet need for treatment of children with musculoskeletal impairment in Malawi

**DOI:** 10.1186/s12887-022-03113-8

**Published:** 2022-01-28

**Authors:** Leonard Banza Ngoie, Eva Dybvik, Geir Hallan, Jan-Erik Gjertsen, Nyengo Mkandawire, Carlos Varela, Sven Young

**Affiliations:** 1 Department of Surgery, Kamuzu Central Hospital, P.O. Box 149, Lilongwe, Malawi; 2 Department of Clinical Medicine, University of Bergen, Bergen, Norway; 3 Centre for International Health, University of Bergen, Bergen, Norway; 4 The Norwegian Arthroplasty Register, Haukeland University Hospital, Bergen, Norway; 5 Department of Orthopaedic Surgery, The Norwegian Arthroplasty Register Haukeland University Hospital, 5021 Bergen, Norway; 6 Department of Orthopaedic Surgery, Haukeland University Hospital, 5021 Bergen, Norway; 7 Department of Surgery, College of Medicine, University of Malawi, Blantyre, Malawi; 8 School of Medicine, Flinders University, Adelaide, Australia

**Keywords:** Musculoskeletal impairment, Childhood disability, Cluster randomized survey, Malawi

## Abstract

**Background:**

More than a billion people globally are living with disability and the prevalence is likely to increase rapidly in the coming years in low- and middle-income countries (LMICs). The vast majority of those living with disability are children residing in LMICs. There is very little reliable data on the epidemiology of musculoskeletal impairments (MSIs) in children and even less is available for Malawi. Previous studies in Malawi on childhood disability and the impact of musculoskeletal impairment (MSI) on the lives of children have been done but on a small scale and have not used disability measurement tools designed for children. Therefore in this study, we aimed to estimate the MSI prevalence, causes, and the treatment need among children aged 16 years or less in Malawi.

**Methods:**

This study was carried out as a national cross sectional survey. Clusters were selected across the whole country through probability proportional to size sampling with an urban/rural and demographic split that matched the national distribution of the population. Clusters were distributed around all 27-mainland districts of Malawi. Population of Malawi was 18.3 million from 2018 estimates, based on age categories we estimated that about 8.9 million were 16 years and younger. MSI diagnosis from our randomized sample was extrapolated to the population of Malawi, confidence limits was calculated using normal approximation.

**Results:**

Of 3792 children aged 16 or less who were enumerated, 3648 (96.2%) were examined and 236 were confirmed to have MSI, giving a prevalence of MSI of 6.5% (CI 5.7–7.3). Extrapolated to the Malawian population this means as many as 576,000 (95% CI 505,000-647,000) children could be living with MSI in Malawi. Overall, 46% of MSIs were due to congenital causes, 34% were neurological in origin, 8.4% were due to trauma, 7.8% were acquired non-traumatic non-infective causes, and 3.4% were due to infection. We estimated a total number of 112,000 **(**80,000-145,000**)** children in need of Prostheses and Orthoses (P&O), 42,000 (22,000-61,000) in need of mobility aids (including 37,000 wheel chairs), 73,000 (47,000-99,000) in need of medication, 59,000 (35,000-82,000) in need of physical therapy, and 20,000 (6000-33,000) children in need of orthopaedic surgery. Low parents’ educational level was one factor associated with an increased risk of MSI.

**Conclusion:**

This survey has uncovered a large burden of MSI among children aged 16 and under in Malawi. The burden of musculoskeletal impairment in Malawi is mostly unattended, revealing a need to scale up both P&O services, physical & occupational therapy, and surgical services in the country.

**Supplementary Information:**

The online version contains supplementary material available at 10.1186/s12887-022-03113-8.

## Introduction

More than a billion people globally are living with disability [1], and the prevalence is likely to increase rapidly in the coming years in low- and middle-income countries (LMICs) [2, 3]. The vast majority of those living with disability are children residing in LMICs [4]. There is increasing evidence that children with disabilities are more likely to come from poorer households, are substantially less likely to attend school and experience poorer health compared to their non-disabled peers [2, 5]. Fewer than 10% of children with disabilities are estimated to attend school in Africa [3]. Disability is argued to have a greater impact on access to education than gender, economic status, or rural/urban residence, and is associated with long-term poverty [6].

In 2003, the WHO published a report highlighting the burden of musculoskeletal conditions in low resource countries [7]. In Africa, conservative estimates state that 11% of the total burden of disease, and 25 million disability-adjusted life years (DALYs) (38/1000 population), are due to surgical conditions [8]. There is very little reliable data on the epidemiology of musculoskeletal impairments (MSIs) in children or the consequences of disability on people with MSI in LMICs [1], and even less is available for Malawi.

A national survey in Rwanda estimated that 2.58% of children under 16 year old have one or more MSIs, of which 43% are moderate or severe [9]. There have been studies in Malawi on childhood disability [10] and impact of musculoskeletal impairment (MSI) in the lives of children [11]. However, these surveys were done on a small scale and did not use disability measurement tools designed for children. Also, there was no verification of self-reported functional limitations by clinical examination.

There is an urgent need to plan appropriate and accessible services for children with MSI in Malawi, and for evidence-based advocacy for funding of this. The authors carried out a national cluster-randomized household survey in 2016, revealing an overall prevalence of MSI of 9.5% (CI 8.9–10.1) in the Malawian population [12]. Using data from this survey, we aimed to estimate the prevalence and the treatment need among children aged 16 years or less in Malawi, and to describe the causes of MSI in Malawian children.

## Methods

This study was based on a cross sectional survey. A list of enumeration areas from the Malawi Census Board for the 2008 national census records was received from the National Statistic Office. Clusters were selected across the whole country through probability proportional to size sampling with an urban/rural and demographic split that matched the national distribution of the population. Clusters were distributed around all 27 mainland districts of Malawi, many at very remote locations with poor roads, and it took the team 2 months, from 1st July to 30th August 2016, to reach all the selected villages by car. Depending on the size of the village, two to four households were randomly selected by spinning a bottle. All individuals present were examined in their households by survey field teams. The standardized selection, interview and examination protocol has been described in detail previously [13] but is outlined here. The definition of MSI that was used was developed from the WHO International Classification of Function (ICF) [14]:

*“ … .a lack of normal structure or function, or an increase in pain or discomfort in the integument, muscles, bone or joints of the body of an individual, that has lasted at least 1 month and which limits function of the musculoskeletal system … ”*

Data collection was done by 32 third-year medical students. They all underwent a 14 days training supervised by two orthopaedic surgeons and two senior orthopaedic clinical officers on how to assess persons with musculoskeletal impairment and the use of the questionnaire and computer tablet. All participants were screened for MSI by asking them seven questions about difficulties using their musculoskeletal system and how long they have had these symptoms. The age cut off of 16 years and under was chosen for ease of comparison to a similar study done in Rwanda [9]. Also, children aged 17 and over are expected to be past or near skeletal bone maturity. According to cultural definitions of adulthood, the latter are usually considered as young adults in our setting.

The screening tool called “Rapid Assessment of Musculoskeletal Impairment” (see Appendix) was developed by researchers and clinical staff at the University of Oxford and the London School of Hygiene and Tropical Medicine for a previous survey in Rwanda, and has been shown to have 99% sensitivity and 97% specificity with interobserver Kappa scores of 0.90 for the diagnostic group [15]. For the youngest (age below 5) household members, the guardian of the child was interviewed. For all participants we also did screen parents’ educational level.

The standardized examination protocol used, if screening questions revealed any MSI, comprised the elements seen in Table 1:

**Table 1:** Standardized interview and examination protocol

Elements	Definition
**Diagnosis**	Diagnosis categorized as: neurological, traumatic, congenital, metabolic, infective, or acquired non-traumatic non-infective. Within these categories an algorithm was created and used to give a specific diagnosis. Up to two diagnoses were permissible per each identified case of MSI [15].
**Severity**	Severity was determined using ICF parameters for the amount of function which was lost through the presence of the impairment. This was classified as “mild”, “moderate” or “severe” [13]. Severity was determined using the parameters for the percentage of function outlined in the WHO reference book International Classification of Functioning (ICF) [14]. A loss of function of 5–24% was mild, 25–49% was moderate and 50–90% was severe
**Treatment received**	Any known treatment given to the participant (medical or other) was recorded
**Treatment needed**	Treatment required by the participants was assessed according to Malawi standard treatment guideline.

All children identified as having impairment in the study were referred to field workers of a community-based rehabilitation (CBR) program for appropriate action such as physiotherapy, prosthetic and orthotic (P&O) devices, or mobility aids. Children found in need of surgery (indication made by the orthopaedic surgeon on the survey team) were referred to a specialist facility for treatment.

Categorical variables was analyzed using a Pearson’s chi square test, continuous variable was tested using independent t-test. The population of Malawi was 18.3 million according to 2018 estimates, and based on the age categories in the same census, we estimated that about 8.9 million were 16 years and younger [12]. MSI diagnosis from our randomized sample was extrapolated to the population of Malawi, confidence limits were calculated using normal approximation. The statistical analyses were performed in the statistical package IBM SPSS Statistics Version 26 (IBM Corp., Armonk, NY, USA) and the statistical package R (http://CRAN.R-project.org). *P*-values less than 0.05 was considered statistically significant.

The approval to conduct the survey was granted by the University of Malawi College of Medicine Research and Ethics Committee (COMREC) and The Regional Committee for Medical and Health Research Ethics (REC West) in Norway. Data collection and handling followed national guidelines in line with the Declaration of Helsinki. Consent to survey the districts and clusters were granted respectively by the District Commissioner and village head for each visited district and cluster. Informed consent to participate in the study was obtained from a parent or guardian before examination. No names of participants, or locations of surveyed households within a cluster were registered in the survey data. Data collectors were allowed to take clinical photographs for teaching and discussion of follow up after a verbal informed consent was granted from the parent or guardian. If a child was found to have a condition needing treatment, a referral to the correct institution was made by the first author, and data kept separate from, and with no linkage to, the study data.

## Results

Of 3792 children aged 16 or less who were enumerated, 3648 (96.2%) were examined. 3.8% of children were not available during the first screening or at one more subsequent visit to the household. Of the 3648 examined participants, 236 were confirmed to have MSI, giving a prevalence of MSI in the 16 and under age group of 6.5% (CI 5.7–7.3). Extrapolated to the Malawian population this means that as many as 576,000 (95% CI 505,000-647,000) children could be living with MSI in Malawi.

Among children found to have MSI, there was no significant difference in the prevalence between children aged 5–16 and those under 5 years of age (*p* = 0.56). Boys were found to have marginally more MSI than girls (7% vs 6%) (*p* = 0.046).

Using the ICF classification the MSIs were classified as moderate or severe in more than 3 out of 4 of the children with MSI. Low parents’ educational level was associated with more MSI among the family members (Table 2).

**Table 2:** Baseline table for the 3648 children

	Total (*N* = 3648)	MSI (*N* = 236)	No MSI (*N* = 3412)	*p*-value
Gender				0.046*
Male	1917	137 (7%)	1780 (93%)	
Female	1731	99 (6%)	1632 (94%)	
Age				0.56
0–5 yr	1109	76 (7%)	1033 (93%)	
5–16 yr	2539	160 (6%)	2379 (94%)	
Location				0.36
Rural	3270	25 (6.5%)	3245 (93.5%)	
Urban	151	11 (7%)	140 (93%)	
Missing	27	0 (0%)	27 (100%)	
Parents educational level				0.005
None	1073	94 (9%)	979 (91%)	
Primary	2240	127 (6%)	2113 (94%)	
Secondary	260	10 (4%)	250 (96%)	
University	25	2 (8%)	23 (92%)	
Missing	50	3 (6%)	47 (94%)	
Severity MSI**				
Mild	55	55 (23.3%) *	0	
Moderate	112	112 (47.5%) *	0	
Severe	69	69 (29.2%) *	0	
Missing	3412	0	3412	

*Percent of the 236 MSI cases

**According to the WHO International Classification of Function

A total of 358 diagnoses were registered in the 236 individuals diagnosed with MSI. The diagnoses are summarized in Table 3 with extrapolated numbers of affected children in Malawi. Overall, 46% of MSIs were due to congenital causes, 34.4% were neurological in origin, 8.4% were due to trauma, 7.8% were acquired non-traumatic non-infective causes and 3.4% were due to infection.

**Table 3:** MSI Diagnosis and Extrapolation to Malawi population

Diagnosis	Number	Total in category (%)	Extrapolated number of that diagnosis in Malawi to nearest 1000 (95% CI)
**Congenital deformity**		165 (46%)	403,000 (343,000–463,000)
Syndactyly	13		32,000 (15,000-49,000)
Polydactyly	29		71,000 (45,000-96,000)
Other UL deformity	19		46,000 (26,000-67,000)
Club foot	19		46,000 (26,000-67,000)
Other LL deformity	28		68,000 (43,000-94,000)
Spine deformity	43		105,000 (74,000-136,000)
Other congenital deformity	14		34,000 (16,000-52,000)
**Trauma**		30 (8.4%)	73,000 (47,000-99,000)
Burn contracture	6		15,000 (3000-26,000)
Fracture non/ malunion	7		17,000 (4000-30,000)
Spine injury	0		
Head injury	2		5000 (0–12,000)
Tendon/nerve injury	6		15,000 (3000-26,000)
Amputation	5		12,000 (2000-23,000)
Joint chronic dislocation	4		10,000 (0–19,000)
Other chronic joint injury	0		
**Neurological**		123 (34.4%)	300,000 (248,000-353,000)
Epilepsy	43		105,000 (74,000-136,000)
Polio (sequelae)	11		27,000 (11,000-43,000)
Para/quadra/Hemiplegia	15		37,000 (18,000-55,000)
Cerebral palsy	46		112,000 (80,000-145,000)
Peripheral nerve palsy	0		
Other neurological MSI	8		20,000 (6000-33,000)
**Infective**		12 (3.4%)	30,000 (13,000-46,000)
Bone infection limb	2		5000 (0–12,000)
Joint infection	3		7000 (0–16,000)
Spine infection	2		5000 (0–12,000)
Soft tissue infection	5		12,000 (2000-23,000)
**Other acquired non-infective non- traumatic**		28 (7.8%)	68,000 (43,000-94,000)
Angular limb deformity	11		27,000 (11,000-43,000)
Joint problem	3		7000 (0–16,000)
Spine pain	0		
Skin/ soft tissue/ bone swelling	2		5000 (0–12,000)
Limb swelling	11		27,000 (11,000-43,000)
Limb pain	0		
Other acquired spine deformity	1		403,000 (343,000–463,000)
**Total**	**358**		

*LL* Lower limbs, *UL* Upper Limbs

**Fig. 1:**
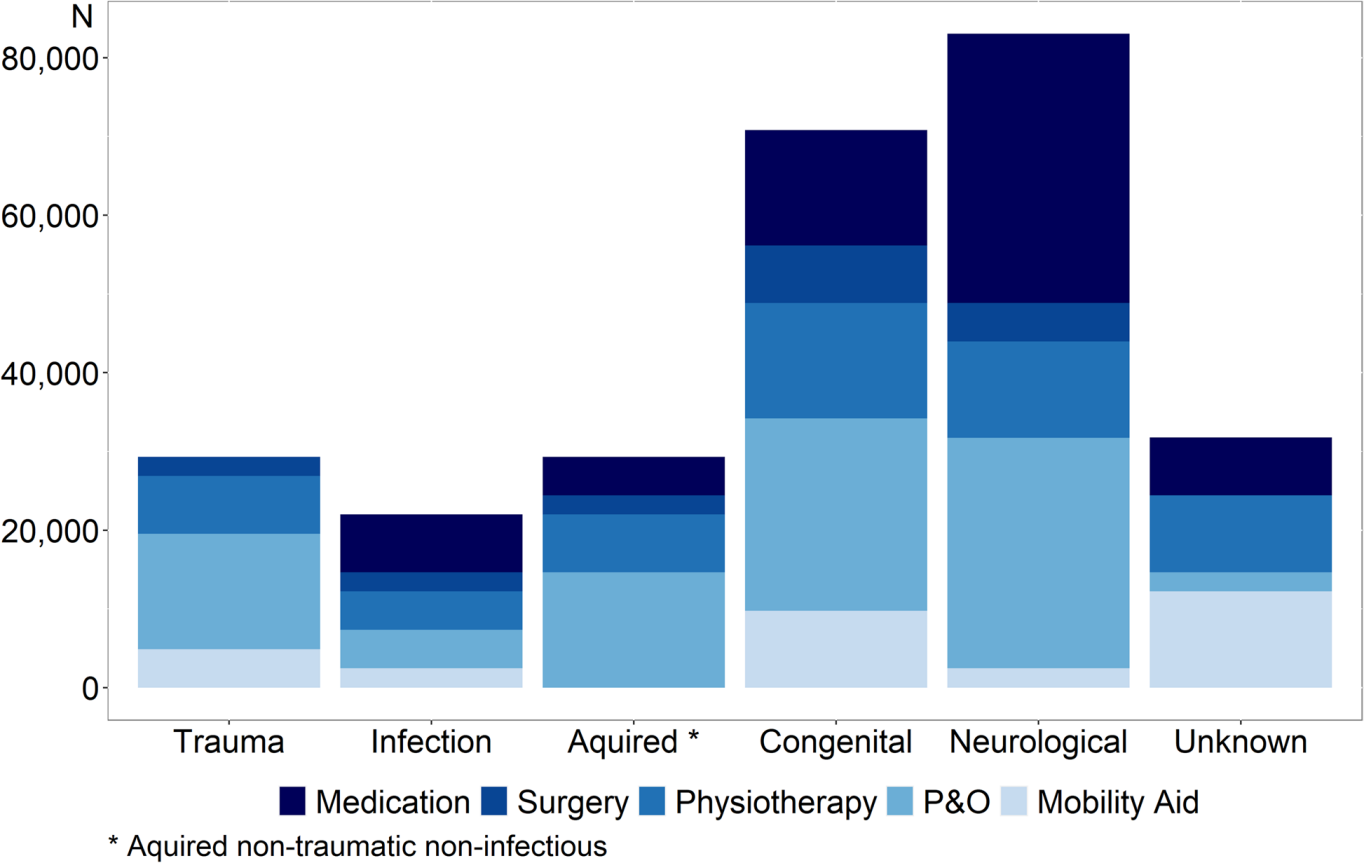
The extrapolated need of treatment for MSI in children in the Malawi population. P&O: Prosthesis and Orthosis

Of the 236 children diagnosed with MSI, 46 were judged to be in need of prosthetic and orthotic (P&O) services, 17 in need of mobility aids (including wheelchairs), 30 in need of medication, 24 in need of physical therapy, and 8 in need of orthopaedic surgery. Extrapolating these findings to the population of Malawi, we estimated a total number of 112,000 **(**80,000-145,000**)** children in need of P&O, 42,000 (22,000-61,000) in need of mobility aids (including 37,000 wheel chairs), 73,000 (47,000-99,000) in need of (mainly anti-epileptic) medication, 59,000 (35,000-82,000) in need of physical therapy, and 20,000 (6000-33,000) children in need of orthopaedic surgery (Fig. 1).

## Discussion

This first countrywide survey of MSI in Malawi revealed that 6.5% of children, or an estimated 576,000 children are living with MSI. Of these, 112,000 children could be in need of P&O devices and 42,000 in need of mobility aids (including 37,000 in need of wheelchairs) to help with ambulation or activities of daily living; 73,000 need medication; 59,000 could benefit from physical therapy, and approximately 20,000 were estimated to benefit from surgery. Currently it is estimated to be only 14 Prosthetists & Orthotists, 200 physiotherapists and physiotherapy assistants, and 15 orthopaedic surgeons in the country.

Studies done on MSI in children in Rwanda and in the Fundong District in North-West Cameroon have shown a prevalence of MSI in children of 16 years and younger of 2.58 and 2.9% respectively [9, 16]. This is less than half the 6.5% proportion of children with MSI in Malawi. The reason of this difference remains unclear. However, extrapolated MSI treatment need estimates in our study have shown that approximately 20,000 children would benefit from surgery in Malawi against 50,000 in Rwanda. The reason for this difference could be that diagnoses and surgical indications in our survey were made by a surgeon, in contrast to the studies from Rwanda and Cameroon, where these were made by physiotherapists.

Most MSI diagnoses in our survey were due to congenital deformities (46%) or were neurological in nature (34.4%). There are few studies to compare our numbers with, but a 5-year audit of all elective orthopaedic operations performed in children at a university hospital in Nigeria showed that congenital limb deformities alone accounted for 35.2% of the diagnoses [17]. A previous study on childhood disability in two districts of Malawi showed that physical impairment (39%) was the commonest impairment type [10], which is in line with this study.

The neurological conditions identified were predominantly cerebral palsy (CP) and epilepsy, which explains the need of medication and P&O devices in those affected children. CP is the most common motor disability in children worldwide, with an estimated prevalence of more than 2 per 1000 in high-resource settings, and a higher prevalence of up to 10 per 1000 reported in low-resource settings [18, 19]. CP is known to be an important and common contributor to childhood disability in low-resource settings [20], as supported by another Malawian study [10].

47.5% of children in our survey had moderate MSI, 29.2% had severe MSI and 23.3% had mild MSI. These findings support those of the World Bank where recent estimates suggest that 5% of all children – 93 million children globally – are living with moderate or severe disability as defined by the World Health Organization (WHO) [2]. Our study also showed that low education in the family was associated with more MSI among the family members. A literature review on disability has suggested that an educated individual with a disability is more likely to better cope with her/his disability than those without education and that chronic health conditions may go improperly monitored by patients who are functionally illiterate and the overall well-being of these individuals may worsen overtime [21].

In common with all population surveys, our study had some limitations. The probability proportional to size sampling, diagnostic tools were limited to history and clinical examination, which restricted the identification of conditions that needed complex investigations. Other limitations were that data on other aspects, such as previous treatment received (some participants were unable to recall their previous treatment) and locations were not collected, and our demographic data were limited. Due to long travel distances in some areas, the call back at a few households where people were unavailable at the initial visit was not achieved. Extrapolations are always associated with some uncertainty and we have been careful to give 95% confidence limits. The study also had several strengths. The response rate of over 96% is very good for a survey in our setting, with the added strength that the study was a nationwide survey with a cluster randomized design to obtain a representative sample of children aged 16 years and younger. The fact that most diagnoses and surgical indications were made by one orthopaedic surgeon, also strengthens the findings and implications in our opinion.

In Malawi, a country with limited resources for health, it is unlikely that the unmet needs for treatment among these children will be met fully in near future. However, the estimates provided in this study should be useful in development of policy and for planning of services in Malawi. With 85% of the Malawian population living in rural areas, access to health and other services may be improved through community-based rehabilitation programs in rural communities. In addition, the development of programs that serve populations at the district level, where needs can be assessed and resources identified, may improve access to preventative services and rehabilitation.

Regarding CP, being one of the most common disabilities among the children, further studies are needed with regard to causes, types, socioeconomic status, education, severity, and other associated medical conditions.

## Conclusion

This survey has uncovered a large burden of MSI among children aged 16 and under in Malawi. Lack of formal education in the family was one factor associated with an increased risk of MSI. The burden of musculoskeletal impairment in Malawi is mostly unattended, revealing a need to scale up prosthetics and orthotics, physical & occupational therapy, and surgical services in the country.

## Supplementary Information

**Additional file 1.** Appendix: Rapid Assessment of Musculoskeletal Impairment (RAM 1 & RAM 2).

## Data Availability

The datasets used and/or analyzed during the current study are available from the corresponding author on reasonable request.
