# Supplementary material for: The unmet need for treatment of children with musculoskeletal impairment in Malawi
Source: BMC Pediatr. 2022 Jan 28;22:67. doi: 10.1186/s12887-022-03113-8 (PMC8796456; doi:10.1186/s12887-022-03113-8)
Supplement: Supplementary file 1 — Additional file 1. Appendix: Rapid Assessment of Musculoskeletal Impairment (RAM 1 & RAM 2). [file 12887_2022_3113_MOESM1_ESM.zip › RAM 2 (English).pdf]

## H. STRUCTURE AND FUNCTION

| Region | Structure affected: Yes | Structure affected: No | Laterality | Nature of change | Magnitude |
|---|---|---|---|---|---|
| 1. Head and Neck | O | O | | | |
| 2. Shoulder region | O | O | | | |
| 3. Upper arm | O | O | | | |
| 4. Elbow Joint | O | O | | | |
| 5. Forearm | O | O | | | |
| 6. Wrist Joint | O | O | | | |
| 7. Hand | O | O | | | |
| 8. Hand/Finger Joints | O | O | | | |
| 9. Whole arm | O | O | | | |
| 10. Pelvis | O | O | | | |
| 11. Hip joint | O | O | | | |
| 12. Thigh | O | O | | | |
| 13. Knee Joint | O | O | | | |
| 14. Lower leg | O | O | | | |
| 15. Ankle Joint | O | O | | | |
| 16. Foot | O | O | | | |
| 17. Foot/Toe Joints | O | O | | | |
| 18. Whole Leg | O | O | | | |
| 19. Trunk | O | O | | | |
| 20. C-spine | O | O | | | |
| 21. T-spine | O | O | | | |
| 22. L-spine | O | O | | | |
| 23. Whole body | O | O | | | |

## I. DIAGNOSTIC CASE CONFIRMATION

Case: O (1)
Not case: O (0)

## J CASE SEVERITY

Case severity: Mild O (1)
Moderate: O (2)
Severe: O (3)

## L. CASE DIAGNOSIS

Diagnosis 1. ____________ Code 1 [ | | | ]

Diagnosis 2 ____________ Code 2 [ | | | ]

## M. TREATMENT INFORMATION

| | Previous Treatment: Yes | Previous Treatment: No | Needed Treatment: Yes | Needed Treatment: No |
|---|---|---|---|---|
| 1. None: | O | O | O | O |
| 2. Medication: | O | O | O | O |
| 3. Plaster/Splintage: | O | O | O | O |
| 4.Physiotherapy | O | O | O | O |
| 5. Special Seating: | O | O | O | O |
| 6. Mobility aid: | O | O | O | O |
| 7. Tricycle: | O | O | O | O |
| 8.Appliance: | O | O | O | O |
| 9. Orthosis | O | O | O | O |
| 10. Prosthesis: | O | O | O | O |
| 11.Wheelchair: | O | O | O | O |
| 12.Surgery: | O | O | O | O |
| 13. Permanent care: | O | O | O | O |
| 14. Traditional medicine: | O | O | O | O |
| 15 Other: | O | O | O | O |
| Specify: | | | | |

## K. DIAGNOSIS DECISION ALGORITHM

Is it congenital? No → Is it due to an infection? No → Is it due to trauma? No → Is it neurological in cause or nature?

Is it congenital? Yes → a. Is it due to an infection? Yes → b. Is it due to trauma? Yes → c. Is it neurological in cause or nature? Yes → d; No → e.

### a. CONGENITAL/GENETIC

UPPER LIMB
- O (01) Polydactyly
- O (02) Syndactyly
- O (03) Other congenital hand deformity
- O (04) Other congenital absence of all or part of upper limb
- O (05) Other congenital abnormality of upper limb

LOWER LIMB
- O (10) Developmental dysplasia of hip
- O (11) Proximal focal femoral deficiency
- O (12) Congenital absence of all or part of tibia
- O (13) Congenital absence of all or part of fibula
- O (14) Other congenital absence of all or part of lower limb
- O (15) Club foot
- O (16) Other congenital abnormality of lower limb

UPPER AND LOWER LIMB
- O (20) Amniotic bands
- O (21) Arthrogryphosis

SPINE
- O (30) Congenital deformity of cervical spine
- O (31) Congenital deformity of thoracolumbar spine

HEAD AND NECK
- O (40) Cleft lip
- O (41) Cleft lip and palate
- O (42) Other congenital deformity of head or face

GENERAL
- O (50) Multiple congenital abnormalities
- O (51) Sickle cell disease
- O (52) Osteogenesis imperfecta
- O (53) Haemophilia
- O (54) Muscular Dystrophy

### b. Infective
- O (01) Joint Infection
- O (02) Bone infection limb
- O (03) Bone infection spine
- O (03) Skin/soft tissue infection/wound

### c. ACQUIRED TRAUMA
- O (01) Burn contracture
- O (10) Fracture non unioin
- O (11) Fracture malunion
- O (12) Spinal injury
- O (13) Head injury
- O (20) Recurrent/chronic dislocation
- O (21) Post traumatic joint stiffness
- O (30) Tendon problem
- O (31) Muscle problem
- O (32) Peripheral nerve problem
- O (40) Amputation
- O (50) Other Trauma

### d. NEUROLOGICAL
- O (01) Epilepsy
- O (02) Leprosy
- O (03) Developmental delay
- O (04) Cerebral palsy - spastic
- O (05) Cerebral palsy - other
- O (06) Paraplegia
- O (07) Hemiplegia
- O (08) Quadriplegia
- O (09) Facial weakness
- O (10) Peripheral nerve palsy
- O (11) Polio
- O (12) Other neurological

### e. ACQUIRED NON-TRAUMATIC
- O (01) Degenerative joint disease
- O (02) Non infective non traumatic joint disease
- O (03) Bow legs
- O (04) Knock knees
- O (05) Other joint deformity
- O (11) Bone tumour (benign or malignant)
- O (21) Skin/Soft tissue tumour
- O (40) Spinal deformity-kyphosis
- O (41) Spinal deformity-lordosis
- O (42) Spinal deformity-scoliosis
- O (43) Spinal pain limiting function
- O (44) TB spine/spine infection
- O (50) Limb pain limiting function
- O (60) Lymphoedema
- O (70) Other acquired non traumatic

### f. NO DIAGNOSIS
O (01) No Diagnosis

## N. WHY I HAVE NOT HAD (FURTHER) TREATMENT

- Unaware of Impairment O (1)
- Believes it to be a curse O (2)
- Services not available or very far O (3)
- No / delayed information about services O (4)
- Cannot afford treatment O (5)
- No one to accompany O (6)
- No time available / other priorities O (7)
- Old age and need not felt O (8)
- Adequate function / need not felt O (9)
- Fear of treatment O (10)
- Not applicable O (11)

**COMPLETION SIGNATURES**
**Physiotherapist**
Name ____________
Signature ____________
Initials [ | | ]

## O. EQ-5D

***1. Mobility***
- I have no problems in walking about O (1)
- I have some problems in walking about O (2)
- I am confined to bed O (3)

***2. Self-care***
- I have no problems with self-care O (1)
- I have some problems washing or dressing O (2)
- I am unable to wash or dress myself O (3)

***3. Usual activities***
- I have no problems with performing my usual activities O (1)
- I have some problem with performing my usual activities O (2)
- I am unable to perform my usual activities O (3)

***4. Pain/discomfort***
- I have no pain or discomfort O (1)
- I have moderate pain or discomfort O (2)
- I have extreme pain or discomfort O (3)

***5. Anxiety/depression***
- I am not anxious or depressed O (1)
- I am moderately anxious or depressed O (2)
- I am extremely anxious or depressed O (3)

Responders own health state today [ | | ]
